# Phylogeographic analysis reveals association of tick-borne pathogen, *Anaplasma marginale*, MSP1a sequences with ecological traits affecting tick vector performance

**DOI:** 10.1186/1741-7007-7-57

**Published:** 2009-09-01

**Authors:** Agustín Estrada-Peña, Victoria Naranjo, Karina Acevedo-Whitehouse, Atilio J Mangold, Katherine M Kocan, José de la Fuente

**Affiliations:** 1 Facultad de Veterinaria, Universidad de Zaragoza, Zaragoza, Spain; 2 Instituto de Investigación en Recursos Cinegéticos IREC (CSIC-UCLM-JCCM), Ciudad Real, Spain; 3 Department of Veterinary Pathobiology, Center for Veterinary Health Sciences, Oklahoma State University, Stillwater, USA; 4 Institute of Zoology, London, UK; 5 Instituto Nacional de Tecnología Agropecuaria, Estación Experimental Agropecuaria Rafaela, Santa Fe, Argentina

## Abstract

**Background:**

The tick-borne pathogen *Anaplasma marginale*, which is endemic worldwide, is the type species of the genus *Anaplasma *(Rickettsiales: Anaplasmataceae). *Rhipicephalus *(*Boophilus*) *microplus *is the most important tick vector of *A. marginale *in tropical and subtropical regions of the world. Despite extensive characterization of the genetic diversity in *A. marginale *geographic strains using major surface protein sequences, little is known about the biogeography and evolution of *A. marginale *and other *Anaplasma *species. For *A. marginale*, MSP1a was shown to be involved in vector-pathogen and host-pathogen interactions and to have evolved under positive selection pressure. The MSP1a of *A. marginale *strains differs in molecular weight because of a variable number of tandem 23-31 amino acid repeats and has proven to be a stable marker of strain identity. While phylogenetic studies of MSP1a repeat sequences have shown evidence of *A. marginale*-tick co-evolution, these studies have not provided phylogeographic information on a global scale because of the high level of MSP1a genetic diversity among geographic strains.

**Results:**

In this study we showed that the phylogeography of *A. marginale *MSP1a sequences is associated with world ecological regions (ecoregions) resulting in different evolutionary pressures and thence MSP1a sequences. The results demonstrated that the MSP1a first (R1) and last (RL) repeats and microsatellite sequences were associated with world ecoregion clusters with specific and different environmental envelopes. The evolution of R1 repeat sequences was found to be under positive selection. It is hypothesized that the driving environmental factors regulating tick populations could act on the selection of different *A. marginale *MSP1a sequence lineages, associated to each ecoregion.

**Conclusion:**

The results reported herein provided the first evidence that the evolution of *A. marginale *was linked to ecological traits affecting tick vector performance. These results suggested that some *A. marginale *strains have evolved under conditions that support pathogen biological transmission by *R. microplus*, under different ecological traits which affect performance of *R. microplus *populations. The evolution of other *A. marginale *strains may be linked to transmission by other tick species or to mechanical transmission in regions where *R. microplus *is currently eradicated. The information derived from this study is fundamental toward understanding the evolution of other vector-borne pathogens.

## Background

The genus *Anaplasma *(Rickettsiales: Anaplasmataceae) contains obligate intracellular organisms found exclusively within membrane-bound inclusions or parasitophorous vacuoles in the cytoplasm of both vertebrate and invertebrate host cells [1,2]. The genus *Anaplasma *includes pathogens of ruminants, *A. marginale*, *A. centrale*, *A. bovis*, and *A. ovis*. Also included in this genus are *A. phagocytophilum*, which infects a wide range of hosts including humans and wild and domesticated animals, and *A. platys *that infects dogs.

To date, most research has been reported for *A. marginale*, the type species for the genus *Anaplasma *[3]. Both cattle and ticks develop persistent infections with *A. marginale *and therefore can serve as reservoirs of infection. *A. marginale *is transmitted horizontally by ixodid ticks including *Rhipicephalus *spp. and *Demacentor *spp. *Rhipicephalus *(*Boophilus*) *microplus *is considered the most important biological vector in tropical and subtropical regions of the world [4]. Transfer of infected blood by biting flies or blood-contaminated fomites effects mechanical transmission of *A. marginale*. The complex developmental cycle of *A. marginale *has been described and shown to be coordinated with the tick feeding cycle [3]. The midgut is the first site of infection, where membrane-bound vacuoles or colonies initially contain reticulated forms that divide by binary fission and subsequently transform into dense forms. Infection of salivary glands and other tissues then occurs which completes the developmental cycle and allows for transmission to susceptible hosts during tick feeding.

Vector-pathogen interactions involve traits from both the vector and the pathogen [5]. Several major surface proteins (MSPs) have been identified and characterized in *A. marginale *[3,5]. MSPs are involved in interactions with both vertebrate and invertebrate hosts [2,3,6-9], and therefore are likely to evolve more rapidly than other genes because they are subjected to selective pressures exerted by host immune systems. The MSP1a of *A. marginale *geographic strains differs in molecular weight due to a variable number of tandem 23-31 amino acid repeats, and the sequence of MSP1a has been shown to be a stable marker for identification of geographic strains [10,11]. Functionally, MSP1a was shown to be an adhesin for bovine erythrocytes and tick cells [12-14]. Tick molecules involved in vector-*A. marginale *interactions were recently identified and functionally characterized [15].

The geographic strains of *A. marginale *are highly variable, as demonstrated by the analysis of MSP1a sequences [7,11]. Such genetic heterogeneity observed among *A. marginale *strains in endemic regions could be explained by cattle movement and maintenance of different genotypes by independent transmission events, due to infection exclusion of *A. marginale *in cattle and ticks which commonly results in the establishment of only one genotype per animal [16-18]. Due to the high degree of sequence variation within most endemic areas, MSP1a sequences have failed to provide phylogeographic information on a global scale [7]. These studies also suggested that multiple introductions of *A. marginale *strains from different geographic locations had occurred in many regions.

The evolutionary history of vector-pathogen interactions can be reflected in the sequence variation of *Anaplasma *MSPs. Previous studies demonstrated that *A. marginale *MSP1a evolved under positive selection [19]. Analysis of *A. marginale *MSP1a repeats provided evidence of tick-pathogen co-evolution [5,6,20], a result that is consistent with the biological function of MSP1a in pathogen transmission by ticks [14]. However, the study of *A. marginale *evolutionary history and tick-pathogen co-evolution has remained elusive because of the extensive genetic diversity of MSP1a sequences.

In this study we analyzed MSP1a repeat and microsatellite sequences in order to provide information on the evolution of *A. marginale *strains by determining their phylogeographic association with world ecological regions (ecoregions) and by testing the effect of different evolutionary pressures associated with the tick vector, mainly *R. microplus*, ecology in these ecoregions.

## Methods

### *Anaplasma marginale *MSP1a repeat sequences

The MSP1a sequences included in this study were obtained from *A. marginale *strains collected worldwide from infected cattle and recently reviewed by de la Fuente *et al*. [11]. The data on MSP1a sequences was updated by searching the Genbank sequence database [21]. The amino acid sequence of the first (R1) and last (RL) MSP1a repeats of 111 *A. marginale *strains were used in this study, from which 39 and 28 unique R1 and RL sequences, respectively, were obtained. For comparison, the sequences of Rn MSP1a repeats, located between R1 and RL, were included in some analyses. MSP1a sequences not included in these studies were from *A. marginale *strains that were not adequately geo-referenced.

### *Anaplasma marginale *MSP1a microsatellite sequences

A microsatellite was located in the MSP1a 5'UTR between the putative Shine-Dalgarno sequence (GTAGG; [6]) and the translation initiation codon (ATG). The structure of the microsatellite (**bold**) was GTAGG **(G/A TTT)m (GT)n **T ATG (Table 1). The microsatellite was sequenced in 115 *A. marginale *strains collected from infected cattle in the USA, Canada, Mexico, Puerto Rico, Argentina, Brazil, Italy, Israel, South Africa and China [11].

**Table 1:** Structure and ecoregion cluster distribution of the *A. marginale *MSP1a microsatellites.

**Genotype**	**Number of strains**	***m***	***n***	**SD-ATG distance (nucleotides)**	**Genotype frequency per ecoregion cluster**			
					
					**1**	**2**	**3**	**4**
A	2	1	7	19	0.00	1.00	0.00	0.00

B	5	1	9	23	1.00	0.00	0.00	0.00

C	7	2	5	19	0.57	0.14	0.29	0.00

D	3	2	6	21	0.33	0.67	0.00	0.00

E	12	2	7	23	0.75	0.00	0.25	0.00

F	3	3	4	21	0.00	0.00	1.00	0.00

G	78	3	5	23	0.15	0.14	0.56	0.14

H	3	3	6	25	0.00	0.67	0.33	0.00

I	NI	4	6	29	---	---	---	---

The MSP1a microsatellite sequences were analyzed in 115 *A. marginale *strains. The microsatellite (sequence **in bold**) was located between the Shine-Dalgarno (SD; sequence in brackets) and the translation initiation codon (ATG) with the structure: GTAGG **(G/A TTT)*m *(GT)*n ***T ATG. The SD-ATG distance was calculated in nucleotides as (4 × *m*) + (2 × *n*) + 1. Abbreviation: NI, not included in the study because the *A. marginale *strain was not adequately geo-referenced.

### World ecoregions and association with *A. marginale *strains

Ecoregions are used herein to classify the world across dynamic environmental factors. We assumed that i) ecoregions could be delineated using quantitative abiotic characters based on well-recognized and repeatable attributes and ii) *A. marginale *strains are associated with each ecoregion and subjected to different environmental conditions that could be analyzed by multivariate geographic clustering [22]. Multivariate geographic clustering involves the use of standardized values for selected environmental conditions in a set of raster maps. Those values serve as coordinates in the environmental data space, in which environmental conditions are further clustered according to their similarities. The feature selected to put together the clusters was the monthly Normalized Difference Vegetation Index (NDVI). NDVI is a variable that reflects vegetation stress, a feature that summarizes information about the ecological background for tick populations [23]. We obtained a 0.1° resolution series of monthly NDVI data for the period 1986-2006. The 12 averaged monthly images were subjected to Principal Components Analysis (PCA) to obtain decomposition into the main axes representing the most significant, non-redundant information. The strongest principal axes were chosen using Cattell's Scree Test [22]. It has been found that the first principal component derived from NDVI typically represents the greenness of the surveyed area [24]. Component 2 is interpreted as a change component, taken to represent a winter/summer seasonality effect. Components 3 and 4 are also essentially seasonal, but represent areas where the timing of green-up is different from that in component 2. Our PCA analysis retained three principal axes, explaining the 92% of total variance. These three axes were related to the mean NDVI values, annual amplitude, and NDVI values in the period May to August, respectively. We then used a hierarchical agglomerative clustering on PCA values to classify multiple geographical areas into a single common set of discrete regions. Mahalanobis distance was used as a measure of dissimilarity and the weighted pair-group average was used as the amalgamation method. A value of 0.05 was used as the cut-off probability for assignment to a given ecoregion. All the procedures adhered to methods previously described [25].

**Figure 1:**
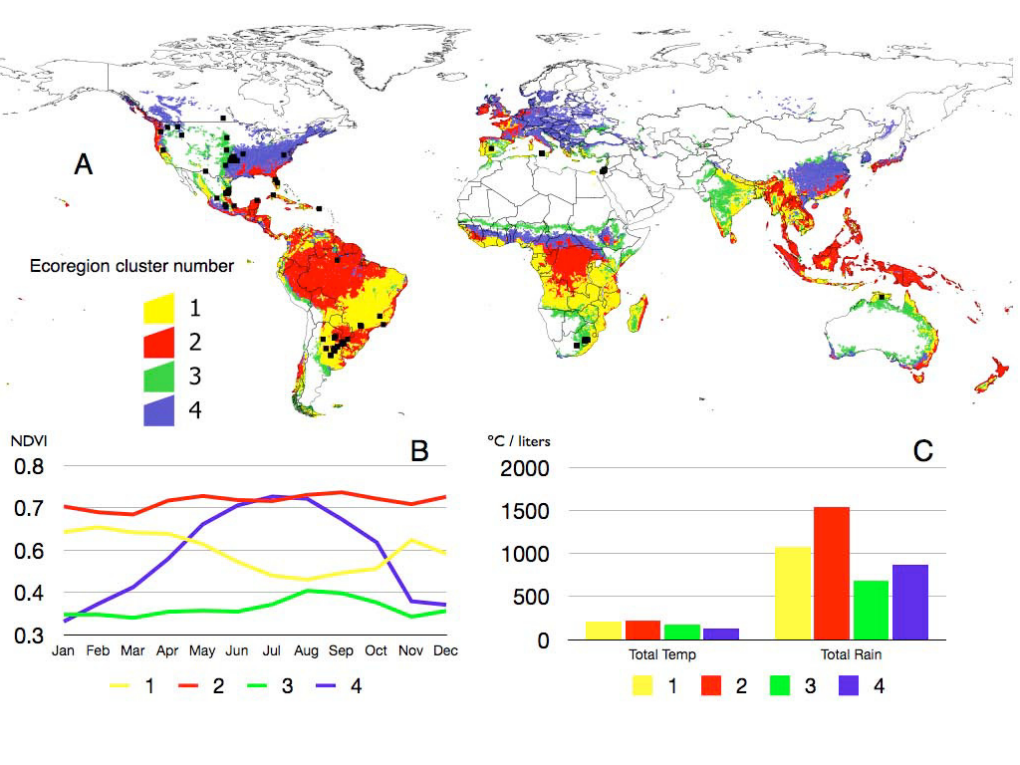
**World ecoregions**. **(A) **Clusters of vegetation features of the world computed through an unsupervised classification on Principal Components Analysis decomposition of monthly normalized difference vegetation index (NDVI) values at a resolution of 0.1°. Small islands were removed to improve the presentation. Only the ecoregions providing most information about *A. marginale *distribution according to a Discriminant Analysis were included, grouped in four main clusters and arbitrarily numbered. Overlying records of *A. marginale *are in black. **(B) **Monthly NDVI values detected for each ecoregion clusters (plotted with the same colors as in A). **(C) **Monthly accumulated temperature and rainfall recorded for each ecoregion cluster.

The decision about the number of ecoregions to retain without any prior detail about the information they contain is a problem to which a solution has not yet been found. The main goal is to define unambiguously the *A. marginale *strains recorded mostly in a single ecoregion cluster and present in the highest number of geographical sites belonging to that cluster. The result is to refine the degree of clustering that gives the optimal degree of association between *A. marginale *strains ('species') and ecoregions ('sites'). This analysis was done using the 'indicator species' method [26], a previously published multivariate statistics procedure to define 'sites' as a function of their faunal composition ('species'). We began the agglomerative process described above with an unrealistic high number of ecoregions. At every step of the agglomerative process, pathogen strains and ecoregions were ordered by a correspondence analysis, and then analyzed using the 'indicator species' method. The procedure runs iteratively, trying to improve the association with further clustering of ecoregions. However, the method does not force a cluster if specifications of cut-off probabilities for ecoregions are violated, and does not assume any *a priori *condition about the geographical range of any cluster. The procedure is only an indicator that stops when an optimum degree of ecoregion clustering collectively explaining the association of *A. marginale *strains with the environment is reached. Such association may be low or high (in the range 0-1) but is the optimal according to the ecoregion features. After the use of these methods we retained a total of four ecoregion clusters (Figure 1) to which *A. marginale *sequences were unambiguously associated, and the rest were discarded because *A. marginale *strains were not isolated there.

### Analysis of *A. marginale *MSP1a repeat sequences

The conserved amino acid sequences of MSP1a R1 and RL repeats reported in *A. marginale *strains from each ecoregion cluster were used to determine the consensus sequence in the cluster. The distances between groups of sequences ascribed to an ecoregion cluster were then computed, both within and between clusters. The hypothesis herein is that highest affinities between R1 and RL isolates are observed when grouped according to the ecoregion to which they are related. An alternative hypothesis is that isolates cluster according to a geographical background. To check for that alternative hypothesis we examined genetic distances with strains arranged into geographical groups. We used only American strains to overcome the undersampling in Africa. They were grouped as Western USA, Eastern USA, Mesoamerica and the Caribbean, and South America, and then distances within and between geographical clusters computed again.

Other analyses involved calculation of the percentage of changes between the consensus MSP1a repeat sequences among different ecoregion clusters as a measure of the genetic distance between *A. marginale *strains in these clusters. Some R1 and RL sequences were found associated with more than one ecoregion cluster. Therefore, the percentage of changes in amino acid composition for every sequence and the consensus sequence in each cluster were computed as a measure of similarity of each sequence and consensus sequence for each cluster. MSP1a repeat sequences were aligned for pairwise comparison and determination of non-synonymous (d_N_) and synonymous (d_S_) substitutions using Mega 4 [27]. The d_N _and d_S _were determined among all pairwise comparisons of MSP1a repeat sequences within each ecoregion cluster, estimated by the method of Nei and Gojobori [28] with the correction for multiple substitutions [29]. The ratio of the mean *d*_N_/*d*_S _was used as an indicator of the level of selection acting on MSP1a repeat sequences.

As an additional test to verify our hypothesis, we performed a Mantel's test. The explanation of the distribution of the strains in terms of environmental variables may be confounded because the variables are intercorrelated among themselves, and so it may be difficult to ascribe causal mechanisms to the environmental variables. Mantel's test is a regression in which the variables are dissimilarity matrices. The operative question is 'do strains that have similar sequences also tend to be similar in terms of the environmental variables?' Therefore we performed a Mantel's test both between a dependent distance matrix (genetic similarities of R1 and RL) and the predictor matrix of geographical distances among strains. A second Mantel's test was done with the same dependent matrix and a predictor dissimilarity matrix based on environmental PCA-derived values. Such correlations will indicate if locations that are closer or locations that are similar environmentally are similar compositionally. Mantel's tests were performed using the Jackard index according to [30].

### Analysis of *A. marginale *MSP1a microsatellite sequences

The extent of genetic differentiation of *A. marginale *strains at the MSP1a microsatellite was assessed within and among ecoregion clusters using an analysis of molecular variance (AMOVA; [31]) and pairwise population *F*_ST _significance tests as implemented in ARLEQUIN, version 3.01 [32]. The statistical significance of fixation indices was tested using a non-parametric permutation approach [33] with 20,000 permutations. Ecoregion clusters for which statistically significant subdivision was not detected were pooled to define groups.

The effect of microsatellite size on MSP1a expression was characterized in *A. marginale *strains Wetumka (OK; Genbank accession number AY010247), Okeechobee (FL; AY010244), Idaho (ID; M32868) and HB-A8 (China; DQ811774) sequences. These strains had microsatellite genotypes G (Wetumka and Okeechobee; distance SD-ATG = 23 nucleotides), C (Idaho; SD-ATG = 19 nucleotides) and I (China; SD-ATG = 29 nucleotides). The *msp1alpha *gene containing promoter sequences active in *Escherichia coli *[6] was amplified using oligonucleotide primers MSP1aP: 5'GCATTACAACGCAACGCTTGAG3' and MSP1a3: 5'GCTTTACGCCGCCGCCTGCGCC3' and cloned into pGEM-T vector (Promega, Madison, WI, USA) as reported previously [20]. Three independent clones for each of the MSP1a constructs were transformed in *E. coli *JM109 cells and grown for 15 to 20 hours at 37°C. Culture volumes of 3 ml were used for RNA and DNA extraction using TriReagent (Sigma, St. Louis, MO, USA) according to manufacturer's instructions. The RNA samples were treated with RNase-free DNase (Invitrogen, Carlsbad, CA, USA) prior to RT-PCR. MSP1a mRNA levels were characterized by real-time RT-PCR using oligonucleotide primers MSP1RT5: 5'ACCAATCGTTGGCAGAAGAG3' and MSP1RT3: 5'ACCTGCTCCCAAAGTAGCAA3' and normalizing against *E. coli *D-1-deoxyxylulose 5-phosphate synthase gene (dxs) [34] and plasmid DNA copy number by *msp1alpha *PCR using the oligonucleotide primers and conditions described above. Real-time RT-PCR was conducted using the iScript One-Step RT-PCR Kit with SYBR Green and an iQ5 thermal cycler (Bio-Rad, Hercules, CA, USA). Control reactions were performed using the same procedures but without reverse transcriptase to test for DNA contamination in the RNA preparations and without RNA added to detect contamination of the PCR reaction. The normalized mRNA levels were compared between different MSP1a constructs using an ANOVA test (*P *= 0.05).

## Results

### *Anaplasma marginale *MSP1a repeat sequences show ecoregion-specific signatures

Ecoregion clusters showed different NDVI, temperature and rainfall values (Figure 1). Ecoregion cluster 1 extended over large areas of central Africa and central South America, primarily Argentina and southern Brazil. It involved a region with medium to high NDVI values with a clear seasonal decrease between June and September. This was the ecoregion with the highest recorded temperature and around 1,000 mm of annual rainfall. Ecoregion cluster 2 included vast areas of the Mesoamerican corridor, northern South America and a small territory of eastern South Africa. It consisted of zones with high NDVI along the year without seasonal variability, temperature values similar to those in ecoregion cluster 1 and rainfall around 1,500 mm/year. Ecoregion cluster 3 extended over central South Africa and scattered parts of southern USA and Mexico, with the lowest NDVI values and little change across the year. This ecoregion displayed lower temperature values and the minimum rainfall. Finally, ecoregion cluster 4 extended over large areas of USA and had a clear NDVI signature, very low between November and March and then rising to reach maximum levels around July. This area was the coldest among all the ecoregion clusters and rainfall was around 800 mm/year.

**Figure 2:**
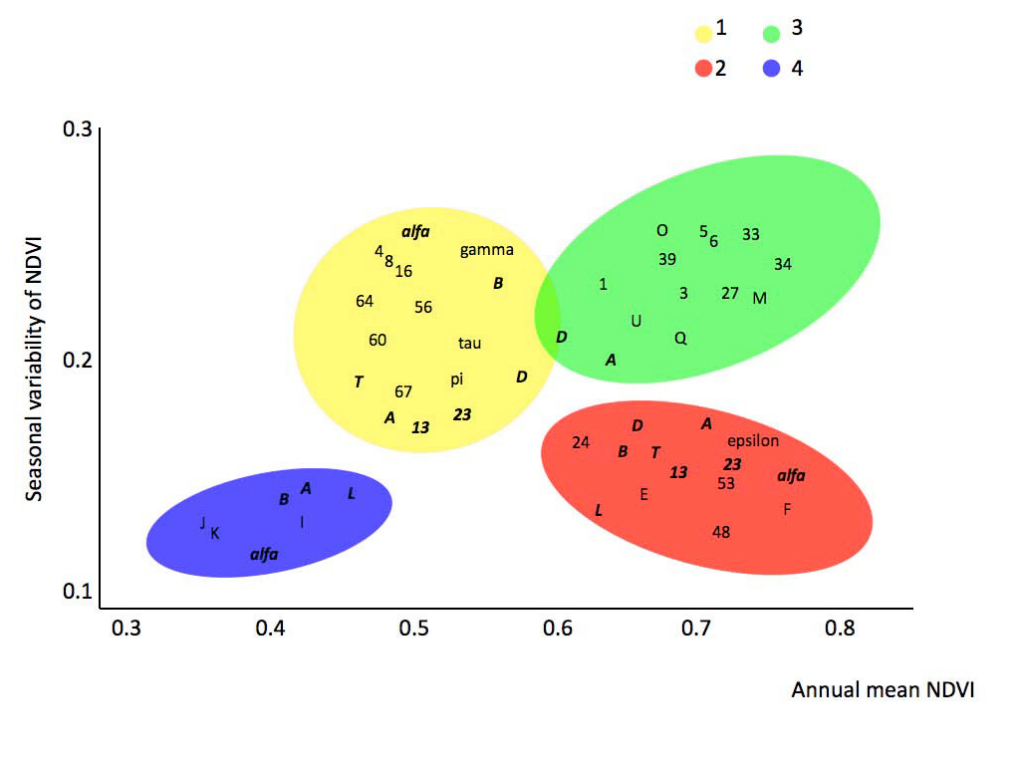
**Associations between *A. marginale *MSP1a R1 repeat sequences and ecoregion clusters, plotted along the values of the first two axes derived from Principal Components Analysis on normalized difference vegetation index (NDVI) time series**. Figure plots the 80% confidence ellipses of the annual mean normalized difference vegetation index (NDVI) and the seasonal variation of NDVI for each ecoregion cluster, as well as the plot of the isolates in the NDVI envelope. Each letter displays the mean position of the records for that strain. Unique sequences for each ecoregion are displayed in plain type. Sequences recorded in more than one ecoregion cluster are displayed in italic bold.

**Figure 3:**
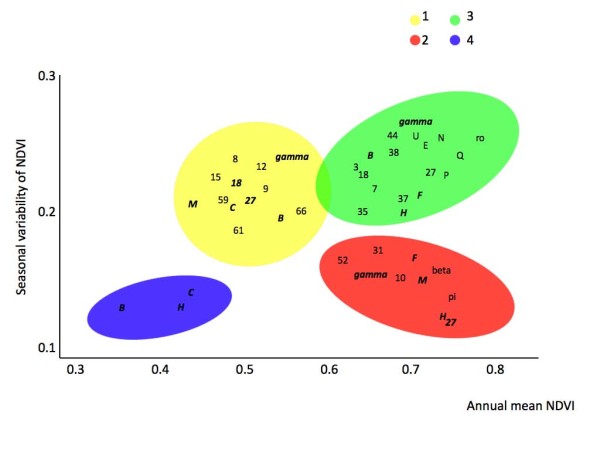
**Associations between *A. marginale *MSP1a RL repeat sequences and ecoregion clusters, plotted along the values of the first two axes derived from Principal Components Analysis on normalized difference vegetation index (NDVI) time series**. Figure plots the 80% confidence ellipses of the annual mean normalized difference vegetation index (NDVI) and the seasonal variation of NDVI, for each ecoregion cluster, as well as the plot of the isolates in the NDVI envelope. Each letter displays the mean position of the records for that strain. Unique sequences for each ecoregion are displayed in plain type. Sequences recorded in more than one ecoregion cluster are displayed in italic bold.

Figures 2 and 3 display the association of the *A. marginale *strains with the four ecoregion clusters. These figures are plotted according to the values of the first two axes derived from PCA on NDVI time series. Figures plot the 80% confidence ellipses of the annual mean NDVI and the seasonal variation of NDVI for each ecoregion cluster, as well as the plot of the isolates in the NDVI envelope. Analysis showed that 77% of MSP1a R1 unique sequences were associated with only one ecoregion cluster (Figure 2). Ten R1 unique sequences (25.6% of the total number of R1 sequences) were reported exclusively in ecoregion cluster 1 and they shared 16 out of 31 amino acids (51.6% of the total number of amino acids; Table 2). Six R1 unique sequences (12.8%) were reported solely in ecoregion cluster 2 with 64.5% identical amino acids. Twelve R1 unique sequences (30.7%) were found only in ecoregion cluster 3, sharing 64.5% of their amino acids. Only three R1 sequences were exclusively associated with ecoregion cluster 4, with 77.4% identical amino acids. All of the *A. marginale *MSP1a R1 sequences within each ecoregion cluster appeared to be under positive selection as shown by d_N_/d_S _indexes of 1.83, 1.61, 1.54 and 1.21 for ecoregion clusters 1 to 4, respectively.

**Table 2:** MSP1a R1 and RL repeat sequences of unique sequences unambiguously associated to only one ecoregion cluster, including the consensus sequence of the isolates of that cluster.

**MSP1a Repeat**	**Unique sequences**	**Consensus sequence**	**Other strains and number of substitutions**
R1/Ecoregion 1	4, 8, 16, 56, 60, 64, 67, gamma, pi, tau	***SSA***QQ*SSV*S*S**AS*SSQ*G--	A(0), B(0), D(1), T(0), 13(1), 23(1), alfa(0)

R1/Ecoregion 2	28, 48, 53, E, F, epsilon	***SS**GQQQESSV***S*-ASTSSQLG--	A(0), B(0), L(1), T(7),13(1), 23(1), alfa(0)

R1/Ecoregion 3	1, 3, 5, 6, 27, 33, 34, 39, M, O, Q, U	**SSSA*GQQQESSV*****QA*TSSQLG--	A(0), D(0)

R1/Ecoregion 4	I, J, K	*D*S*A*GQQQESSVSSQS*QASTSSQLG--	A(0), B(0), L(0), alfa(0)

RL/Ecoregion 1	8, 9, 12, 15, 59, 61, 66	***SSA**QQQES*V*SQS**ASTSSQ*G--	B(0), C(0), M(0), 18(0), 27(0), gamma(0)

RL/Ecoregion 2	10, 31, 52, pi, beta	*DSSSA**QQQ*S*V*S*S*-ASTSSQLG--	F(0), H(0), M(0), 27(0), gamma(0)

RL/Ecoregion 3	3, 7, 35, 37, 38, 44, E, N, P, Q, U, ro	*DSSSAS*QQQESS**S*S*QA**S*Q*G--	B(1), F(0), H,18(0), gamma(0)

RL/Ecoregion 4	none		B, C, H

Other strains recorded from more than one ecoregion cluster are included in the last column, stating the number of substitutions in their amino acid sequences as compared with the consensus sequence in that ecoregion cluster (in parentheses).

Differences were found among the R1 sequences at each ecoregion cluster. Comparison of consensus sequences between clusters 1 and 2 revealed 22.5% of amino acid differences. Ecoregion clusters 1 and 3 differed by 25% of their consensus sequences while ecoregion clusters 2 and 3 had only 19% of different amino acids in their R1 sequences. Five R1 sequences, T, 13, 23, D and L were found simultaneously in two of the ecoregion clusters (Figure 2, Table 2). Details of their similitude with the consensus sequences of each ecoregion cluster are included in Table 2. Three R1 sequences, A, B and alpha appeared associated with different ecoregions. Pairwise comparisons between *A. marginale *R1 sequences demonstrated that genetic distances were lower within than between ecoregion clusters (Table 3). However, when strains were compared as clustered geographically, intra-cluster distances were much higher, revealing a clear heterogeneity between geographic strains and rejecting the hypothesis of a pure geographical association. Mantel's test on R1 sequences was 0.82 (*P *< 0.001) when applied to ecoregion clusters using only unique sequences. Mantel's test on R1 sequences dropped to 0.72 (*P *< 0.005) when every sequence in the ecoregion clusters was included. The same test provided a value of 0.31 (*P *= 0.145) for the distances matrix based on geographical association of strains.

**Table 3:** Top table shows the genetic distances among and between the MSP1a R1 repeat sequences reported on each ecoregion cluster.

**Ecoregion**	**Cluster 1**	**Cluster 2**	**Cluster 3**	**Cluster 4**
**Cluster 1**	**0.212**	0.311	0.319	0.337
**Cluster 2**		**0.199**	0.321	0.371
**Cluster 3**			**0.175**	0.214
**Cluster 4**				**0.156**
**Geographical cluster**	**Western USA**	**Eastern USA**	**Mesoamerican+ Caribbean**	**South America**
**Western USA**	**0.418**	0.399	0.514	0.519
**Eastern USA**		**0.329**	0.388	0.501
**Mesoamerican+**			**0.454**	0.455
**Caribbean**				
**South America**				**0.521**

Bottom table displays the genetic distances among and between the MSP1a R1 repeat sequences clustered on a geographical basis.

Numbers in bold represent the distance between the MSP1a R1 repeat sequences contained within each ecoregion cluster.

Discriminant analysis showed that 74.8% of MSP1a RL sequences were found associated with one ecoregion cluster only (Figure 3, Table 2). Seven RL sequences (25% of the total number of RL sequences) were reported exclusively in ecoregion cluster 1, with 61.2% identical amino acids. Five RL sequences (17.8%) were reported solely in ecoregion cluster 2 with 64.5% identical amino acids. Twelve RL sequences (32%) were found only in ecoregion cluster 3, sharing 61.2% of their amino acids. No RL unique sequences were found in ecoregion cluster 4. The differences in RL consensus sequences between ecoregion clusters 1 and 2, 2 and 3, and 1 and 3 were 16%, 26% and 32%, respectively. In contrast to *A. marginale *MSP1a R1 repeat sequences, the RL sequences within each ecoregion cluster did not appear to be under positive selection as shown by *d*_N_/*d*_S _indexes of 0.89, 0.79 and 0.77 for ecoregion clusters 1 to 3, respectively. Mantel's test on RL sequences was 0.87 (*P *< 0.001) when applied to ecoregion clusters using only unique sequences. This value dropped to 0.82 (*P *< 0.001) when every strain in the ecoregion clusters was included in the analysis. The same test provided a value of 0.27 (*P *= 0.208) for the distances matrix based on geographical association of strains. When the MSP1a Rn repeats, located between R1 and RL were analyzed, a total of 30, 9, 17 and 3 sequences were ascribed to ecoregion clusters 1 to 4, respectively. The Rn consensus sequences contained 14 (45.2%), 17 (54.9%), 17 (54.9%) and 24 (77.4%) identical amino acids in ecoregions clusters 1 to 4, respectively. The number of R1, Rn and RL repeat sequences varied between ecoregion clusters without a clear pattern.

### *Anaplasma marginale *MSP1a microsatellite sequences have ecoregion-specific signatures and affect gene expression in *Escherichia coli*

The analysis of the MSP1a microsatellite sequences resulted in nine different genotypes among *A. marginale *strains (Table 1). The different microsatellite sequences produced SD-ATG distances of between 19 and 29 nucleotides, but the predominant distance was 23 nucleotides in all regions (Table 1).

The analysis of MSP1a microsatellite sequences showed phylogeographic clustering of some genotypes (Table 1). However, only three of the eight microsatellite genotypes included in the analysis were exclusive to a particular ecoregion cluster: genotype A to ecoregion cluster 2, genoytpe B to ecoregion cluster 1 and genotype F to ecoregion cluster 3. A significant genetic differentiation was found between ecoregion clusters for MSP1a microsatellite genotype frequencies, with overall *F*_ST _= 0.16 (*P *< 0.0001). The ecoregion clusters 1 and 2 were clearly distinct from the other ecoregion clusters, and the largest *F*_ST _value (0.28) was found between ecoregion clusters 1 and 4 while significant genetic differences were not found between ecoregion clusters 3 and 4 (Table 4). To control for potential confounding effects of geography, we investigated genetic differentiation between broad geographic regions. This showed that genetic differentiation is mostly dependent on ecoregion-based clustering of the strains, with the highest *F*_ST _value being 0.07 for South America and the Mediterranean (see Table 4). The AMOVA showed that 84% of the variance accounted for within-ecoregion cluster differences, while a geographic-based AMOVA showed that within-population variation explained less (73%) of the variance (Table 5).

**Table 4:** Ecoregion and geographic cluster pairwise FST significance tests (bottom half of each section) and *P*-values (top half of each section) of *A. marginale *MSP1a microsatellites.

**Ecological**	**Ecoregion 1**	**Ecoregion 2**	**Ecoregion 3**	**Ecoregion 4**
**Ecoregion 1**	-	0.019 ± 0.001	0.000 ± 0.000	0.001 ± 0.000
**Ecoregion 2**	0.09	-	0.027 ± 0.001	0.032 ± 0.001
**Ecoregion 3**	0.22	0.07	-	0.312 ± 0.003
**Ecoregion 4**	0.28	0.15	0.016 (NS)	-

**Geographical**	**North America**	**South America**	**Mediterranean**	**South Africa**

**North America**	-	0.324 ± 0.003	0.016 ± 0.001	0.232 ± 0.003
**South America**	0.004 (NS)	-	0.036 ± 0.001	0.452 ± 0.003
**Mediterranean**	0.06	0.07	-	0.101 ± 0.002
**South Africa**	0.01 (NS)	-0.01 (NS)	0.07 (NS)	-

Abbreviation: NS, not significant.

**Table 5:** Hierarchical analysis of molecular variance (AMOVA) for MSP1a microsatellites.

**Source of variation**	**d. f**.	**Sum of squares**	**Variance components**	**Percent variation**
**Ecological**				
Among ecoregions	3	3.99	0.04	16.31
Within ecoregions	109	24.49	0.22	83.69
Total	112	28.48	0.27	---
**Geographic**				
Among continents	2	1.16	-0.08	-35.70
Among populations within continents	6	8.76	0.15	63.18
Within populations	104	18.57	0.18	72.52
Total	112	28.48	0.25	---

Table shows Wright's fixation index, *F*_ST_. Statistical significance was calculated from 20,000 random permutations. Fixation indices and *P*-values: (Ecological) *F*_ST _= 0.16 (*P *= 0.00 ± 0.00), (Geographic) *F*_ST _= 0.29 (*P *= 0.00 ± 0.00).

The effect of MSP1a microsatellite size on gene expression was analyzed using the sequence derived from Wetumka, Okeechobee, Idaho and HB-A8 *A. marginale *strains (Table 6). The results showed that MSP1a expression was lower in the construct containing the Idaho strain-derived MSP1a sequence with the lowest SD-ATG distance of 19 nucleotides, while differences were not observed between constructs containing the MSP1a sequences from Wetumka, Okeechobee and HB-A8 *A. marginale *strains with SD-ATG distances of 23 and 29 nucleotides (Table 6).

**Table 6:** Effect of microsatellite genotype on the expression of *A. marginale *MSP1a in *E. coli*.

***A. marginale *strain**	**Microsatellite genotype**	**SD-ATG distance (nucleotides)**	**Normalized MSP1a mRNA levels (arbitrary units)**
Idaho, ID	C	19	1.57 ± 0.29 (a)

Wetumka, OK	G	23	2.31 ± 0.27 (b)

Okeechobee, FL	G	23	2.13 ± 0.0 (b)

HB-A8, China	I	29	2.39 ± 0.19 (b)

MSP1a mRNA levels were analyzed by real-time RT-PCR in three independent clones for each of the MSP1a constructs transformed in *E. coli *JM109. MSP1a mRNA levels were normalized against *E. coli dxs *gene and plasmid DNA copy number by *msp1α *PCR. Normalyzed MSP1a mRNA levels were represented in arbitrary units as average ± SD and compared between different constructs using an ANOVA test (different letters denote significant differences; *P *< 0.02).

## Discussion

Vector-borne pathogens have evolved molecular mechanisms of vector-pathogen interactions that involve genetic traits of both the vector and the pathogen [5,35]. For the tick-borne pathogen *A. marginale*, recent studies have characterized tick and pathogen-derived genes that are involved in tick-pathogen interactions [2,3,6-9,15]. Phylogenetic studies using tick and/or pathogen-derived genetic markers have contributed to our understanding of the evolution of *A. marginale *strains and tick-pathogen relationships [5,6,20]. However, the impact of these studies has been limited by the genetic diversity of genes involved in tick-pathogen interactions and thus are likely to reflect pathogen evolution and tick-pathogen relationships [5].

In this study we took a different approach to characterize the evolution of *A. marginale *strains. This is the first study to use remotely sensed vegetation features as a surrogate of an environmental envelope to which genetic variability and structure of a single pathogen is associated. Biogeographic research seeks to identify the processes structuring organism diversity at a variety of geographic and taxonomic scales [36]. Remote sensing is being used increasingly as a tool to discover ecological traits through definite signatures. NDVI is a measure of the vegetation stress, thus a time series of NDVI values over a region reflects the seasonal cycle of vegetation as a surrogate of the seasonal variation in climate. NDVI and other climate features are commonly used to detect ecologically suitable areas for some pathogens and their vectors [37-39]. For example, Randolph and Rogers [40] indicated that climate has directed and constrained the evolution of flaviviruses of the TBE group. Six flaviviruses (SSEV, WTBEV, Russian TBEV, OHFV, and Kyasanur forest virus) fall within a distinct eco-climatic space defined by factors derived from thermal and moisture conditions. Herein we showed that *A. marginale *MSP1a R1 repeats evolved under positive selection, were associated with specific ecoregion clusters and were not arranged according to geographical features. The different evolutionary pressures operating over different MSP1a repeats was demonstrated previously [6], but the possibility of using the MSP1a R1 repeat as a biogeographical marker has only been suggested [5]. In contrast, MSP1a RL repeat sequences, while still linked to a similar set of ecoregion clusters, did not evolve under positive selection. Consequently, RL repeat sequences were not good genetic markers for the characterization of *A. marginale *biogeography and evolution.

Analysis of MSP1a microsatellite sequences demonstrated that *A. marginale *strains are associated with specific ecoregion clusters, thus corroborating the results obtained with repeat sequences. These results may have a functional significance. It has been shown that the SD-ATG distance between the ribosome binding site (Shine-Dalgarno sequence) and the translation initiation codon affects gene expression in prokaryotes [41]. Little is known about the regulation of gene expression in *A. marginale *[6]. However, as shown here in *E. coli*, the length of the MSP1a microsatellite could affect the expression of MSP1a, which varies during *A. marginale *multiplication in both tick cells and bovine erythrocytes, thus affecting pathogen infection and transmission [42]. Since MSP1a repeats and microsatellites are unambiguously associated to ecoregion clusters, these results suggested a new factor that may affect the efficiency by which different *A. marginale *strains are transmitted under different environmental conditions.

*A. marginale *is an obligate intracellular parasite, which alternates between the tick vector and the vertebrate host. Our hypothesis was that the link between pathogen strains and definite portions of the environmental envelope could reflect the effects of climate on the tick vector. Temperature and rainfall, which are indirectly captured by the specific signatures of NDVI, are the main factors affecting the ecology and population dynamics of tick species [43] and these operate at critical levels of selection of tick populations, selecting also specific strains of the pathogen. This framework is further obscured by the 'noise' produced by invasive events of the pathogen (by cattle movement or other factors), or by selection of strains transmitted mechanically in areas where ticks are eradicated by acaricide application, contributing to the absence of total consistence between ecoregion clusters and strains.

Adequate reports exist about distribution, seasonal dynamics and abundance of *R. microplus *populations in the study area, allowing a direct comparison with results presented herein. Ecoregion cluster 1 contained the R1 repeats with the lowest percentage of conserved amino acids and the highest positive selection pressure, in areas with high temperature and medium rainfall. *R. microplus *ticks are common in these areas and a strict seasonality in tick population dynamics has been reported, allowing for a high selection of tick populations due to winter mortality [44]. Ecoregion cluster 2 contained sites with constant high temperature and rainfall. In these sites, *R. microplus *ticks are abundant throughout the year without marked seasonality and climate is not a limiting factor in tick mortality [44,45]. In ecoregion cluster 3, tick populations suffer drastic limitations in effectiveness because of low and inadequate rainfall [46], and this high selection pressure on tick populations might be adverse for pathogen transmission and selection. The R1 repeat sequences in ecoregion clusters 2 and 3 had higher number of conserved amino acids and lower positive selection pressure when compared with R1 sequences in ecoregion cluster 1. Finally, the R1 repeat sequences ascribed to ecoregion cluster 4 had the highest percentage of conserved amino acids and the lowest positive selection pressure, recorded only in sites where *R. microplus *ticks are absent because of the low yearly temperature and thus other tick species act as vectors of *A. marginale *[18]. The analysis of MSP1a microsatellite sequences also supported differences among all ecoregion clusters, except for ecoregion clusters 3 and 4 where *R. microplus *has low prevalence or is absent.

Some R1 repeat sequences such as A, B, D and alpha as well as microsatellite genotypes C-D, G and H were present across several ecoregion clusters. These sequences appeared in *A. marginale *strains collected in zones where *R. microplus *ticks are common (ecoregion clusters 1 and 2) and in sites, such as central Argentina and southern parts of the USA, where *R. microplus *has been prevalent in the past but has been eradicated [46]. Additionally, these sequences were also found in sites where other tick vectors such as *Dermacentor *spp. are prevalent [18]. These R1 repeats and microsatellite sequences could have evolved from ancestor pathogen strains transmitted by *R. microplus *as the main vector, and then evolved under lower selection pressure, due to pathogen transmission by other tick species or mechanically. The presence of these sequences in sites where *R. microplus *has been historically absent (that is, north-western USA) and now adapted to transmission by *Dermacentor *spp. ticks, could be interpreted as invasive events. The results reported here showed that lowest selection pressure exist in sites where *Dermacentor *spp. ticks are the main biological vectors or where mechanical transmission is predominant because of eradication of *R. microplus*. Therefore, R1 repeats are evolving under high selection pressure only in sites where *R. microplus *is the main vector and is subjected to selection because of climate constraints. This hypothesis did not explain the absence of MSP1a genetic diversity in Australia. Analysis of four *A. marginale *strains in Australia revealed the presence of a single repeat type 8 [11]. We would expect evolution of *A. marginale *MSP1a towards different repeat sequences, sharing the consensus sequence found in ecoregion cluster 1, into which R1 type 8 is ascribed, even in the case of a single invasive event. Reasons accounting for such a lack of diversity are currently unknown, but the combined pressure exerted by tick population structure [47,48] the *A. centrale *vaccine, acaricide treatments and cattle movement for pathogen and tick control may have impacted on *A. marginale *genetic diversity in Australia [48].

*A. marginale *exclusively infects cattle and wild ruminants [2]. Such high host specificity may results in a relatively low impact of vertebrate host factors on the evolution of *A. marginale *strains, thus leaving tick-pathogen interactions as the main contributing factor affecting its biogeography and evolutionary history. However, as previously discussed, cattle movement may have contributed to the genetic diversity of *A. marginale *strains worldwide [11]. Nevertheless, the results reported herein may be relevant in studying the evolution of other vector-borne pathogens. Many vector-borne pathogens, such as some *Babesia*, *Theileria*, *Rickettsia*, *Ehrlichia *and *Plasmodium *species, are also highly host-specific [49] and vector-pathogen interactions may play a crucial role in their evolution and biogeography [35].

## Conclusion

The results reported herein provided the first evidence that the evolution of *A. marginale *MSP1a repeat and microsatellite sequences was linked to environmental traits, and that strains are not geographically related. Different evolutionary pressures acting on *A. marginale *were found associated with zones where climate and rainfall affect presence, abundance and dynamics of vector populations. We hypothesized that some *A. marginale *strains evolved under conditions of pathogen biological transmission by *R. microplus*, while others may be linked to transmission by other tick species or to mechanical transmission in regions where *R. microplus *is currently absent. The procedures outlined herein could be fundamental toward studying the evolution of other vector-borne pathogens.

## Authors' contributions

AEP, JF and AJM conceived and designed the study and conducted MSP1a repeat sequence analysis. AEP conducted ecoregion analysis and clustering. VN, KAW and JF conducted MSP1a expression and microsatellite sequence analyses. AEP, KAW KMK, and JF contributed to drafting the manuscript. All authors read and approved the final manuscript.
